# Nanosuspension of Foeniculum Vulgare Promotes Accelerated Sensory and Motor Function Recovery after Sciatic Nerve Injury

**DOI:** 10.3390/metabo13030391

**Published:** 2023-03-07

**Authors:** Majed A. Bajaber, Ghulam Hussain, Tahir Farooq, Razia Noreen, Muhammad Ibrahim, Huma Umbreen, Shaheera Batool, Kanwal Rehman, Arruje Hameed, Muhammad Fayyaz Farid, Tanzeela Khalid

**Affiliations:** 1 Chemistry Department, Faculty of Science, King Khalid University, Abha 61413, Saudi Arabia; 2 Neurochemicalbiology and Genetics Laboratory (NGL), Department of Physiology, Government College University Faisalabad, Faisalabad 38000, Pakistan; 3 Department of Applied Chemistry, Government College University, Faisalabad 38000, Pakistan; 4 Department of Biochemistry, Government College University Faisalabad, Faisalabad 38000, Pakistan; 5 Department of Nutritional Sciences, Government College University Faisalabad, Faisalabad 38000, Pakistan; 6 Department of Biochemistry, CMH Institute of Medical Sciences Multan, Multan 60000, Pakistan; 7 Department of Pharmacy, The Women University Multan, Multan 60000, Pakistan

**Keywords:** nanosuspension, bioavailability, nerve regeneration, oxidants, secondary metabolites

## Abstract

The seed extract of *Foeniculum vulgare* (FV) was used for the preparation of a nanosuspension (NS) with an enhanced bioavailability of phytoconstituents. Subsequently, it was employed as a potent source of polyphenols, such as quercetin and kaempferol, to accelerate the regeneration and recovery of motor and sensory function in injured nerves. The NS was administered through daily gauging as NS1 (0.5 mg/mL) and NS2 (15 mg/mL), at a dose rate of 2 g/kg body weight until the end of the study. The NS-mediated retrieval of motor functions was studied by evaluating muscle grip strength and the sciatic functional index. The recovery of sensory functions was assessed by the hotplate test. Several well-integrated biochemical pathways mediate the recovery of function and the regeneration of nerves under controlled blood glucose and oxidative stress. Consequently, the NS-treated groups were screened for blood glucose, total antioxidant capacity (TAC), and total oxidant status (TOS) compared to the control. The NS administration showed a significant potential to enhance the recuperation of motor and sensory functions. Moreover, the oxidative stress was kept under check as a result of NS treatments to facilitate neuronal generation. Thus, the nanoformulation of FV with polyphenolic contents accelerated the reclamation of motor and sensory function after nerve lesion.

## 1. Introduction

Peripheral nerve injuries generally result in the loss of motor and sensory functioning of nerves, often leading to permanent physical disability. The functional loss arises from the degenerate axons and the damage to the integrity of Schwann cells (SCs). A number of integrated cellular mechanisms coordinate axonal regenerations for functional retrieval after nerve injury [1,2]. A growth-permissive environment is created by the non-neuronal SCs for injured axons. A number of structural and molecular changes appear in distal and proximal stumps of the injured nerves, as a mechanistic approach to support the regeneration of axons [3]. Injured neurons cause the downregulation of mRNAs involved in neurotransmissions and the upregulation of those required for regeneration. As an immediate response, the growth-associated proteins (GAP)-43, tubulin, and actin, are upregulated to provide a conducive environment for nerve regeneration [4]. Thus, the injured nerves possess the inherent potential for regeneration, generally a complicated and slow process [5,6,7]. A progressive decline is observed in upregulated biomachinery that slows down the regeneration of axons, prolonging the process of nerve repair and not allowing the full retrieval of functions. Therefore, different drugs are administered to accelerate the full reclamation of sensory and motor functions in injured nerves. However, they are often unable to support the required levels of the growth-permissive environment for functional recovery and nerve regeneration [8,9].

Over the last few decades, medicinal plants have received global attention as an alternative source of bioactive molecules with a broad spectrum of biological activities. Natural medicines have become attractive choices for the management of various diseases owing to their cheaper availability and harmless nature. Many dietary plants and herbs have been evaluated for their elemental ingredients and composition of phytoconstituents to predict their potency against various diseases and possible toxic risks [10,11,12,13]. Accordingly, there has always been a search for new therapeutics capable of achieving a complete recovery of damaged nerves, with low or manageable toxic effects. Therefore, plant metabolites have become attractive alternatives as neurotherapeutics owing to their multidimensional chemistry, safer nature, and better efficacy profiles [14,15]. A number of plant-based secondary metabolites have been identified as effective drugs against various diseases, including neurological disorders. They have been studied as potential leads for modern drug development due to their diverse chemistry and venerable nature for further derivatization [16,17,18]. The natural molecules, including taxol, capsaicin, quercetin, and curcumin, have shown anticancer, anti-HCV, antimicrobial, and antioxidant properties, and they are recognized as potential candidates for the development of alternative medicines. Likewise, various phytocompounds have proved their effectiveness against several neurological diseases, such as Parkinson’s disease, Alzheimer’s disease, and Huntington’s disease [19,20,21].

*Foeniculum vulgare* (FV), belonging to the Apiaceae family, is known for anti-inflammatory, antioxidant and hepatoprotective properties [22,23,24]. Phytoconstituents with antioxidant potential are potent to accelerate functional recovery after nerve injury [25,26]. Recent studies have demonstrated that oral administration of crude and methanolic exact of FV could effectively accelerate the regaining of function after sciatic nerve injury [27,28]. The plant metabolites generally show low bioavailability due to their pH-sensitive chemical nature and poor adsorption across biological membranes [29,30]. The recent developments in nanotechnology have helped in the preparation of the nanoformulation of drugs with the sustained release of bioactive molecules for longer periods. Accordingly, the nanosuspension formulation enhances the bioavailability of poorly soluble phytoconstituents with controlled delivery, thus improving their bioactivity profiles [31,32,33].

Keeping the above facts in mind, the study was planned to prepare a nanosuspension of FV with an enhanced bioavailability of functional molecules. The prepared nanosuspension of FV was evaluated for its capacity to accelerate the regeneration process, with complete recuperation of sensory and motor functioning after induced nerve lesion in a mouse model.

## 2. Material and Methods

### 2.1. Animals

Healthy male albino mice (age: 8–9 weeks; weight: 25–27 g) were obtained from a local vendor. Under optimum humidity and a controlled environment, they were subjected to a dark/light cycle of 12 h at 26 ± 1 °C in the housing facility at the Department of Physiology, Government College University, Faisalabad. During the study period, they were sub-divided into two nanosuspension-treated groups (*n* = 6/group) and a control group (*n* = 6) and fed separately in clean cages, with one subject/cage. During this experiment, the behavior-related studies were performed at specified times during the light cycle, and days −1, −3, or −5 represent the pre-injury period. 

### 2.2. Collection and Processing of Foeniculum vulgare

The *FV* seeds were purchased from the local market, shade dried, and ground into powdered form. Then, 50 g of seed powder was added into a mixture of methanol/water (300 mL (1:1)) and stirred for 12 h. Subsequently, the filtrate was dried under vacuum with the help of a rotary evaporator, followed by heating in an oven for 2 h at 40 °C [34,35].

### 2.3. Preparation of Foeniculum vulgare Nanosuspensions 

First, FV seed extract (20 mg), citric acid (50 mg), and tocopheryl polyethylene glycol succinate, as a stabilizer (30 mg), were dissolved in ethyl acetate and stirred constantly for 1 h. Then, a vacuum rotary evaporation apparatus was used to evaporate the organic solvent. After that, an aqueous carbonate was added to the organic acid phase. Consequently, the nanosuspension (NS) was obtained after the rapid generation of CO_2_ bubbles [36].

### 2.4. Characterization of Nanosuspension

**Figure 1:**
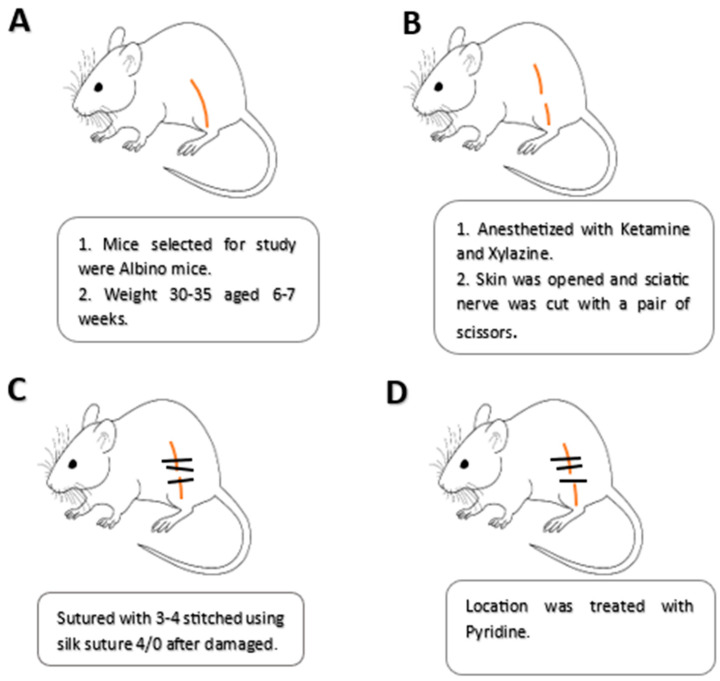
Characterization of nanosuspension of *F. vulgare* (**A**); TEM analysis (**B**); size distribution (**C**); FTIR analysis (**D**).

The prepared NS was characterized using TEM, which confirmed the average diameter of the NPs at around 45 ± 3 nm. The FTIR analyses confirmed the presence of polyphenolics and flavonoids in the prepared NS sample (Figure 1). The FTIR spectrum was used to identify the functional groups of the active components present in the plant samples, based on the peak values in the region of IR radiation. The FTIR spectroscopic results were in agreement with those of previous studies. The methanolic seed extract of Foeniculum vulgare revealed the presence of phenols, phenolic glycosides, flavonoids, etc. Quercetin and kaempferol are the commonly known polyphenols present in the methanolic extract of FV [37,38].

### 2.5. Supplementation of FV Nanosuspension 

The as-prepared SN1 and SN2 were administered through daily gauging as NS1 (0.5 mg/mL) and NS2 (15 mg/mL), at the daily dose rate of 2 g/kg body weight [39]. They were supplemented until the end of the experiment. On a daily basis, the food intake and average body mass were measured simultaneously [40].

### 2.6. Induction of Sciatic Nerve Crush Injury 

**Scheme 1:**
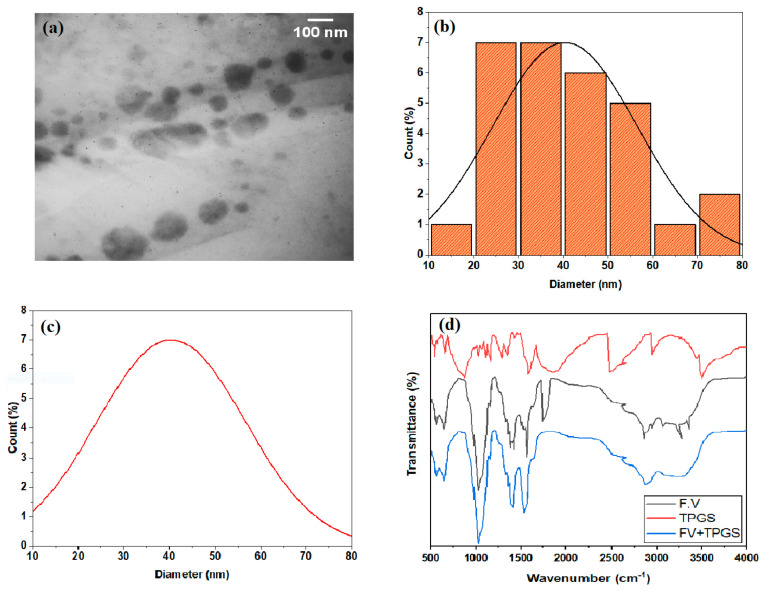
Induction of sciatic nerve crush injury.

In order to study the effects of the prepared nanosuspensions on the functional retrieval of a nerve injury, surgical methods were followed to induce sciatic nerve lesions in well-acclimatized mice of the previously mentioned groups [41]. In each subject, the right thigh muscles were exposed with an incision after intraperitoneal injection of a mixture of ketamine (70 mg/kg) and xylazine (5 mg/kg). Thereafter, the sciatic nerve was subjected to compression, followed by skin suturing and disinfection. At the end of the trial, the mice were subjected to decapitation for the harvesting of tissues and collection of blood samples (Scheme 1).

Histopathology is a technique for detecting tissue changes in the tibialis anterior muscles. The tissue is fixed after placing it in 10% formalin. After 24–48 h, the sample is transferred to a PBS (phosphate buffer) solution. Following fixation, tissues are dehydrated using various alcohol concentrations. We used 70% alcohol for 4–8 h, 85% alcohol for 4 h, 95% alcohol for 5 h, absolute alcohol for 1.5 h, and absolute alcohol II for 1.5 h. In the clearing phase, xylene I (25–30 min) and xylene II (20–30 min) were used as dehydrating agents. For a few hours, the tissues were infiltrated with embedding fluid (molten paraffin wax) at 50–600 °C; paraffin blocks were used to embed filtered tissues. Using a rotary microtome, the tissues were thinly sectioned (5 m thickness) (Lecia RM2125 RTS, Rawalpindi, Pakistan). After sectioning, thin tissue slices were collected onto a glass slide. The slides were stained with hematoxylin and eosin stain. Images were observed at 40× magnification using a light microscope (XSZ 107BN, Beijing, China) and captured using a digital camera (Optikam B1, Optika, Ponteranica, Italy) [27].

### 2.7. Behavior-Related Studies

#### 2.7.1. Sciatic Functional Index 

Well-established methods were followed for the measurement of SFI, an index to study the regaining of motor functions after nerve injury [28,42]. The mice were permitted to walk on a wooden track to a given mark. Subsequently, the paw prints were followed for the measurement of SFI using the following equation:

$$\mathrm{SFI}=\left(-38.5\times \frac{\mathrm{EPL}-\mathrm{NPL}}{\mathrm{NPL}}\right)+\left(109.5\times \frac{\mathrm{ETS}-\mathrm{NTS}}{\mathrm{NTS}}\right)+\left(13.3\times \frac{\mathrm{EIT}-\mathrm{NIT}}{\mathrm{NIT}}\right)-8.8$$

Here,

NPL = normal print length, EPL = experimental print length, NTS = normal toe spread, ETS = experimental toe spread, NIT = normal intermediate toe, and EIT = experimental intermediate toe. 

#### 2.7.2. Hot Plate Test 

The regaining of sensory functioning in injured nerves was analyzed by performing the known hot plate test. The recovering subjects were allowed to stand on hot plate (54 ± 1 °C) until showing any response. The mean response time was recorded as the hot plate latency (HPL) period, after three measurements [27].

#### 2.7.3. Assessment of Grip Strength of Muscle

According to the accepted procedure, the grip strength meter was used to access the regaining of muscle grip in the recovering mice. Each subject tried to stop the unintentional backward movements until the withdrawing force weakened their grip. The peak of pulling force after concorded readings were noted for the ipsilateral, contralateral, and hind limbs [41].

### 2.8. Biochemical Studies

#### 2.8.1. Total Antioxidant Capacity

A known TAC method was followed to analyze the antioxidant potential of the recovering subjects after injury. The method involves Trolox solution as a standard against serum samples; the 2,2′-azinobis 3 ethylbenzothiazoline-6-sulfonate (ABTS) generates free radicals when incubated with hydrogen peroxide [43]. The samples were analyzed using a chemistry analyzer (Biosystem, BTS-330), and TAC was expressed as mmol Trolox equiv/L [44].

#### 2.8.2. Total Oxidant Status (TOS)

The TOS was studied in NS-treated and control subjects following an established procedure. The method involves the conversion of Fe^2+^ into Fe^3+^ using an oxidant in an acidic environment. The oxidation status in the serum samples was tracked using xylenol orange using a semi-automated chemistry analyzer (Biosystem, BTS-330) [45].

#### 2.8.3. Random Blood Glucose

In recovering mice, the glucose levels were analyzed in blood samples using a glucometer (Accu-chek) [28]. The optimum glycemic level mediates underlying biochemical pathways for accelerated nerve regeneration and the retrieval of functions. 

## 3. Results

### 3.1. Effects of NS-Treatments on Body Mass and Food Consumption 

**Figure 2:**
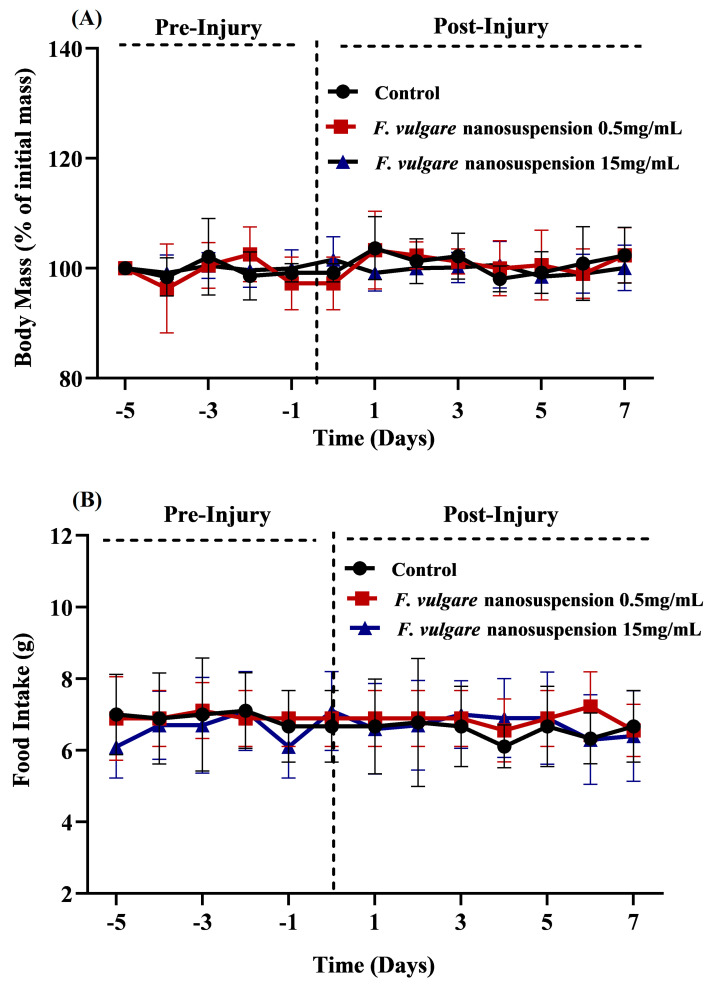
Impact of *F. vulgare* on the mice (**A**) body mass and (**B**) food intake.

The food intake and body mass were studied in the NS-treated and control groups during the course of the experiment (after and before the sciatic nerve injury) (Figure 2A,B). A nonsignificant difference on all days suggested that the administration of nanosuspensions did not disturb the eating behavior of the mice. This was attributed to an acceptable taste, smell, and granularity of the NS. Further, the subjects exhibited a steady increase in body mass, with the nonsignificant difference suggesting that NS supplementation did not influence the metabolic activities.

### 3.2. Effects of NS-Treatments on Regaining of Motor Functions and Muscle Strength 

**Figure 3:**
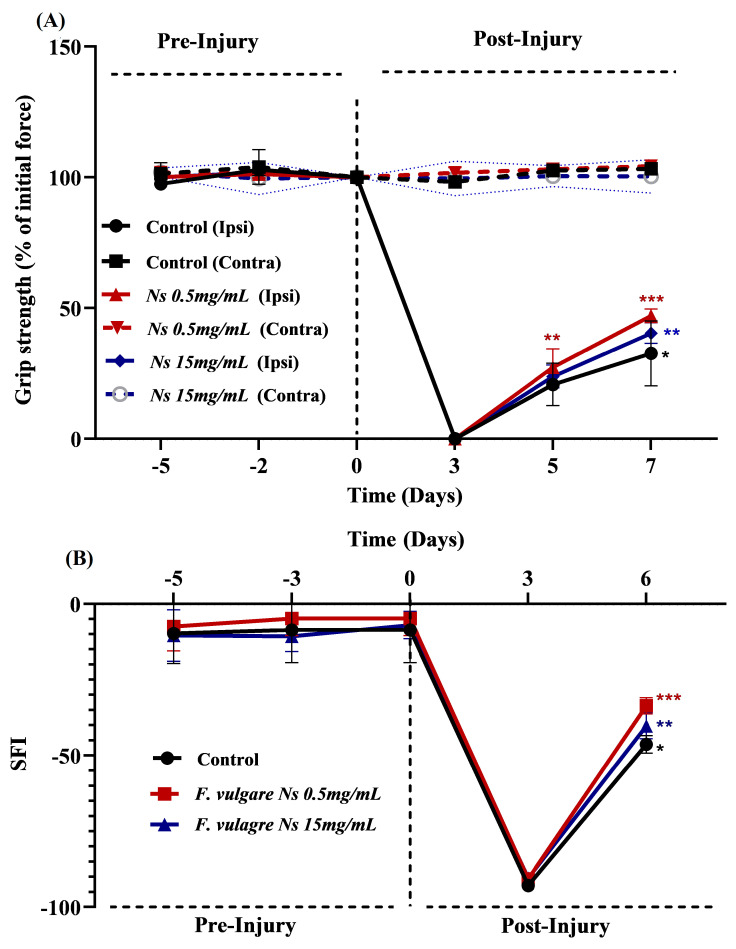
Influence of *F. vulgare* on (**A**) muscle grip strength and (**B**) SFI following sciatic nerve lesion. *, **, *** represents the significance of results/data.

The regaining of motor functions in the subjects was analyzed according to muscle grip strength and the measurement of SFI. The nanosuspension-treated groups displayed a significantly quick recovery of grip strength compared to the control group (Figure 3A). However, in comparison to NS2, the NSI supplementation caused a highly significant and positive change in muscle strength from days 5 to 7 post-injury. In both NS-treated groups, a substantial enhancement of SFI was observed on post-injury days 3 to 6 compared to that of the control group (Figure 3B). Again, a maximum increasing impact was induced by NS1. So, an improved SFI represents a faster functional retrieval of motor functions after induced injury.

### 3.3. Effects of NS-Treatments on Regaining of Sensory Functions

**Figure 4:**
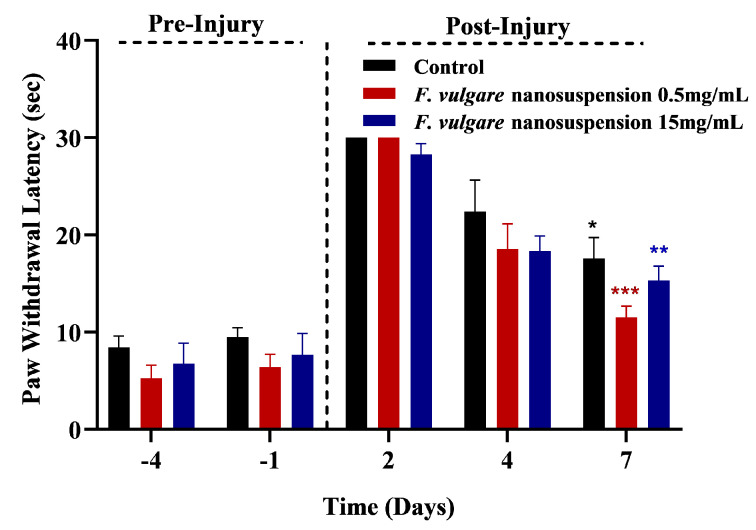
Effects of *F. vulgare* on recovery of sensory function following sciatic nerve lesion. *, **, *** represents the significance of results/data.

Nerve injuries are a common cause of losing motor and sensory functions in suffering subjects. During this experiment, the recovery of sensation was analyzed in treated and non-treated groups using hotplate tests, before and after the induction of injury. Both of the NS-treated groups displayed a significantly faster regaining of sensory function compared to the control group. However, the maximum positive effect was induced by NS1 treatment, as reflected by an enhanced sensation at day 7 post-injury (Figure 4).

### 3.4. Effects of NS-Treatments on Glycemic Level 

**Figure 5:**
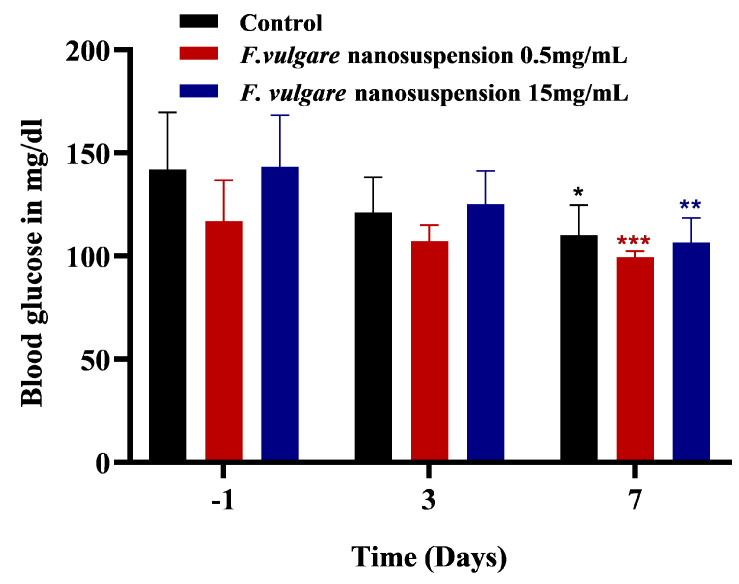
Effects of *F. vulgare* on blood glucose level. *, **, *** represents the significance of results/data.

On day 7 post-injury, a significant controlled glycemic level was observed in both NS-treated groups (Figure 5). However, a well-controlled glycemic level was noted in NS1 treated subjects.

### 3.5. Effects of NS-Treatments on Oxidative Stress 

**Figure 6:**
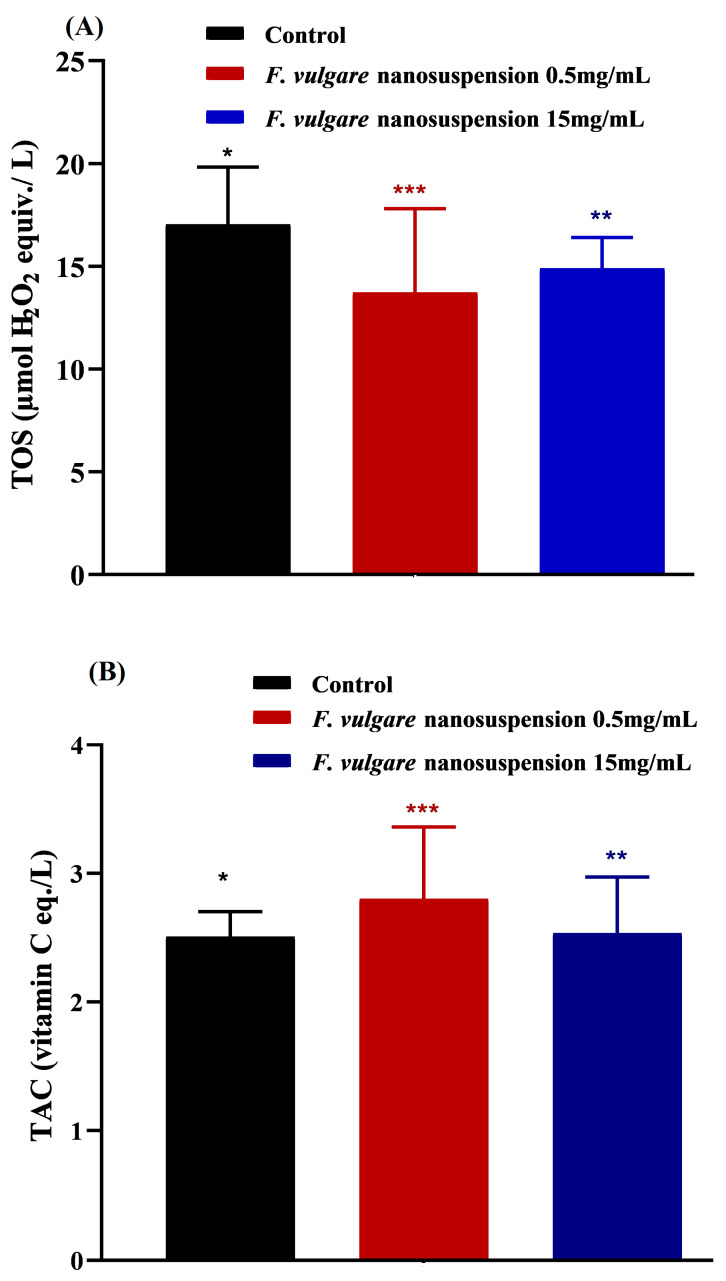
Effects of *F. vulgare* on (**A**) TOS and (**B**) TAC following sciatic nerve lesion. *, **, *** represents the significance of results/data.

The regeneration and functional retrieval processes are greatly influenced by the development of local oxidative stress after nerve lesion. In this connection, the TOS and TAC were studied in NS-treated and untreated subjects to evaluate their antioxidant effects (Figure 6).

Compared to the untreated control, both NS1 and NS2 treated groups demonstrated significantly higher TAC levels, while maximum increasing impact was observed after NS1 treatment. Similarly, the NS treatments significantly controlled the oxidant status compared to the control. Again, the best results were observed in the NS1 treated groups. Thus, the administration of the prepared nanosuspension regulated the oxidant status by controlling antioxidants after nerve lesion.

### 3.6. Effects of Nanosuspension on Muscle Histology

**Figure 7:**
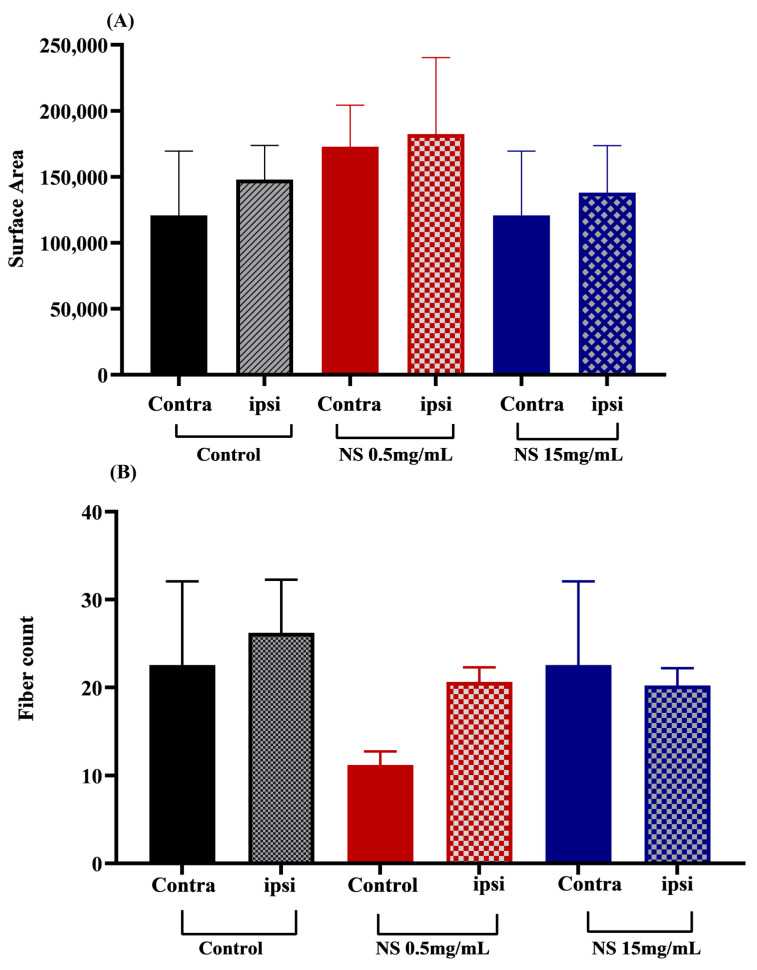
Effects of *F. vulgare* on (**A**) surface area and (**B**) fiber count contralateral v/s ipsilateral in tibialis muscles.

**Figure 8:**
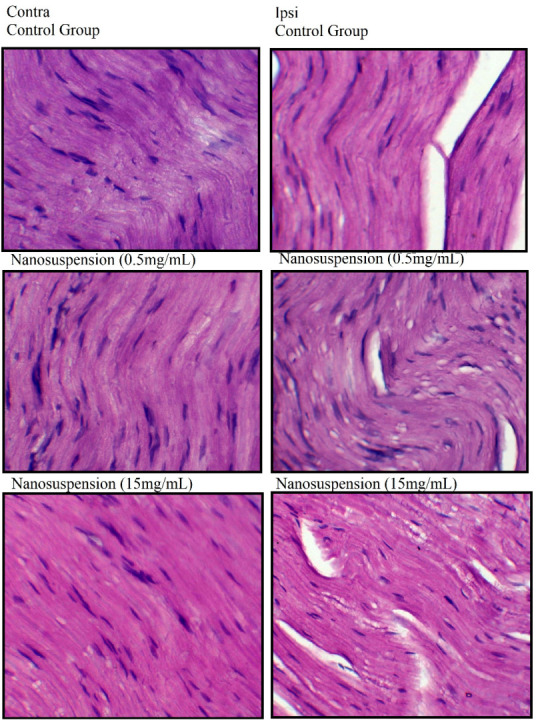
Impact of *F. vulgare* on muscular histology (Images taken as 40× magnification).

Generally, injured nerves show interrupted electrical signal transduction, and the prolongation of the situation results in muscle atrophy. The surface area and fiber count studies were performed for the histopathological comparisons of ipsilateral and contralateral sides in the NS-treated and control groups (Figure 7 and Figure 8). The NS-treatments caused a significant improvement in the reclamation of muscular morphology by reducing dystrophy after induced nerve injury. 

## 4. Discussion

Nerve injuries cause the imbalance of ROS homeostasis, which disturbs an array of biochemical processes and sub-cellular signaling, resulting in the delay of functional recovery and nerve regeneration. This influences the excessive production of tumor necrosis factor alpha (TNF-α) and pro-inflammatory cytokines such as interleukin-6 (IL-6) and IL-1. It further activates inducible nitric oxide synthase (iNOS) and the expression of cyclooxygenase-2 (COX-2). All such changes in the microenvironment cause mitochondrial dysfunction, abnormal neuronal activity, and neurotransmitter disturbance [46]. The prolonged oxidative stress leads to the damaging of macromolecules, such as like lipids, proteins, and DNA, consequently causing cell death. Hence, ROS-induced cellular apoptosis and prolonged inflammatory responses result in demyelination and axonal degeneration, leading to the neurodegeneration process, delaying nerve regeneration and functional recovery after injury [47]. Polyphenols, owing to their anti-inflammatory and antioxidant potential, could support and modulate the regeneration process after PNI. Therefore, polyphenols could be good candidates as complementary diet substitutes for supporting peripheral nerve regeneration [48]. Over the centuries, plant metabolites have established their worth as potent sustainable sources and key targets for drug development. FV is traditionally known as a nutrient supplement containing a variety of phytochemicals, including kaempferol, quercetin, and other polyphenols with diverse chemistry and high biological potential. The FV seed extract was used to prepare nanosuspensions, anticipating the high bioavailability of phytoconstituents for accelerated regeneration and functional retrieval after nerve lesion. Structurally diverse metabolites were made available to assess their potential to accelerate recovery after induced nerve injury. The administration of the as-prepared nanosuspension produced no significant change in the eating behavior of the recovering subjects. Both of the NS treatments caused a significant increase in body mass, suggesting the nutritive values of bioavailable phytoconstituents potent enough to modulate metabolic activities as a stress-counteracting approach [49,50,51]. The plant-based polyphenols have the capacity to alter lipid and carbohydrate metabolism by interacting with gut microbiota. In doing so, they improve the metabolism of adipose tissue and attenuate dyslipidemia and hyperglycemia [52,53]. On day 7 post-injury, a maximum increment, with a non-significant difference, was observed as a result of NS1 treatments. The *FV* seeds are enriched with reserves of proteins, glycosides, flavonoids, and a variety of minerals. Therefore, they are credited for having a positive influence on the body weight of NS-treated subjects [22,54].

The grip strength studies revealed the protective role of NS treatments on the regaining of motor functions in injured nerves. The subjects treated with NS1 showed significantly higher recovery of grip strength from days 5 to 7 post-injury. The subjects in the untreated group were found to show the slowest regaining of motor functions until the end of the trial. The observed trend signified the positive role of FV-based phyotmolecules arbitrating the recovery of motor functions following PNI. Earlier studies also showed the positive role of FV phytoconstituents in the recovery of sensory and motor functioning in mice [55,56]. The natural polyphenols modulate mitochondrial energies and activate the longevity-related protein sirtuin 1 (SIRT 1) as a stress-countering measure in skeletal muscles [57]. The polyphenolic supplementation provides protection against mitochondrial dysfunction and prevents functional impairment and muscular atrophy [58]. A significant improvement in SFI was induced after NS treatments. Again, the maximum increasing impact was observed after NS1 treatment. It represented the available metabolites as a potent factor for an accelerated retrieval of motor functioning in injured nerves. Polyphenolics could regulate apoptosis and improve motor functions after sciatic nerve injury. Actually, they regulate the apoptotic signaling pathway by controlling the expression of some vital proteins [59]. The phenolic compounds, such as isoquercitin and quercitin, exhibited antiapoptotic effects and improved motor functioning after nerve injuries. They could control the myelin function and the cAMP signaling pathway, upregulating the genes involved in axonal growth. They improved the retrieval of motor functions and reduced the muscle atrophy in the mouse model [60,61,62].

Further, the recovery of sensory functions in NS-treated and untreated subjects was studied using the paw withdrawal latency time in the hotplate tests. On day 7 post-injury, the NS treatments induced significant improvements in the regaining of sensory functions. The NS1 treatments provided a relatively quick retrieval in this regard. The recovery of sensory functions suggested the worth of available metabolites as a vital factor for nerve regeneration and functional recovery. The natural polyphenols are known to modulate the TRP channels for the recovery of thermosensitivity in injured nerves [63,64].

Generally, the regeneration process and the regaining of functions in injured nerves depend on oxidation stress. A number of physiological functions and underlying biochemical pathways regulate the production of oxidants to maintain oxidative stress at an optimum level in biological systems, including nerve cells. A well-controlled oxidative status, with a high level of antioxidant capacity, facilitates an accelerated recovery after nerve lesions. Therefore, TOS and TAC status provides a picture of the oxidation state and probable mechanisms involved in the recovery and regeneration process. In this study, the administration of prepared NS triggered a substantial increase in TAC compared to that of the control. Again, NS1 exhibited higher levels of TAC than the NS2-treated group. Similarly, the SN administration induced a significant reduction in TOS, representing a controlled production of oxidants. Hence, a controlled TOS and an improved TAC in the NS-treated groups ensured an optimum level of oxidation stress, supporting functional retrieval after nerve lesion. The phospholipid architecture of cellular membranes is disrupted when the polyunsaturated fatty acids undergo peroxidation due to the excessive generation of free radicals. Further, some key metabolic enzymes such as Na^+^/K^+^-ATPase lose their functions due to the unavoidable interference of products of lipid peroxidation. Such physio-biochemical disruptions delay the axonal growth and regeneration process after nerve injuries. The polyphenolics such as kaempferol and quercetin, the major phytoconstituents of FV, contain known antioxidants with the capacity to scavenge reactive oxygen and nitrogen species. They modulate antioxidant defense systems to regulate the production of oxidants at the injury site [48,65]. Additionally, quercetin and its derivatives coordinate with the JAK2/STAT3 signaling pathway and increase brain-derived neurotrophic factor (BDNF) to regulate cell proliferation and neuronal recovery [66]. Kaempferol and its derivatives control the expression of potent genes and activate proteins involved in antioxidant machinery for the regulation of oxidative stress, favoring the growth and regeneration process [67,68].

The FV-based metabolites provided protection against oxidants and thus are capable of facilitating the regeneration process [69,70,71]. A number of studies have evaluated polyphenols as potent metabolites to accelerate recovery of motor and sensory functions in injured nerves [48,72]. Thus, the enrichment of FV-based flavonoids and polyphenols played a crucial role in regulating oxidant status and boosting the reclamation process after induced nerve lesions. The phytoconstituents of FV are significantly effective to control blood glucose levels, which coordinate with several biochemical processes involved in the regeneration process and retrieval of senses after injury [56,73]. The secondary metabolites of FV, including polyphenols, flavonoids, and polysaccharides, are vital for the regulation of metabolic activities and help to keep blood glycemic levels under control [74,75]. Phenolic compounds have a known positive role in the controlling of blood glucose. They regulate glycemic levels by increasing carbohydrate absorption and by boosting the sensitivity and secretion of insulin. Quercetin could coordinate with a myriad of signaling pathways and factors such as NRF2, AKT, AMPK, and TNFα to maintain blood glycemic levels [76]. Kaempferol activates insulin secretions, suppresses caspase 3 activities, and improves the expression of AMP-related proteins. It can induce the production of new glucose transporters, as well as activate PI3K pathways and protein kinase C, to enhance glucose uptake [77].

Nerve injuries significantly reduce the expression of proteins, such as peripheral myelin protein 22 (Pmp 22) and myelin-associated glycoprotein (Mag), which are vital for the development and protection of myelin sheath. In PNS, Schwann cells control the expression of Mag, and Pmp22 mediates the development and protection of the myelin sheath. Therefore, the number of layers and the thickness of the myelin sheath is significantly reduced as a result of sciatic nerve injury [78,79]. The histopathological comparisons of the ipsilateral and contralateral sides in the NS-treated and control groups validated the results of the behavioral analysis. The surface area and fiber count studies revealed a significant reduction in muscular dystrophy. Further, the NS-mediated optimization of the oxidation state and glycemic levels modulated the regeneration process, as indicated by the retention of normal muscle area and muscular shape. However, the NS-treatments were found to effectively boost the recovery at a lower dose level, as plant extracts often show toxicity when administered at higher concentrations [80]. Quercetin, a potent phenolic antioxidant compound, activates the Akt and Nrf2 pathways and suppresses the ATF-6, NF-kB, and caspase 3 pathways to shield peripheral nerve impairment [81]. According to an earlier report, quercetin administration showed growth-promoting effects, causing a significant increase in the density of myelinated fibers in the regeneration process after PNI [82]. Quercetin applications favored nerve fiber remyelination and Schwann cell proliferation by inhibiting cell apoptosis and oxidative stress. It promoted axonal regeneration by increasing the myelinated fibers. Another study suggested that quercetin administration enhanced the number of nerve fibers and the thickness of myelin sheath [61].

NS administration caused a significant improvement in sensory functions, muscle strength, and body mass in recovering subjects after induced nerve injury. They also increase TAC and reduced TOS, for the regulation of oxidative stress at optimum levels. Further, a positive impact on blood glucose levels illustrated their vital role in an accelerated recovery after PNI. Therefore, the administration of FV-NS at low concentrations produced far better results than the use of simple extracts for the recovery of peripheral nerve injury The NS containing polyphenols such as quercetin and kaempferol controlled oxidative stress, induced anti-apoptotic and anti-inflammatory effects, and facilitated the nerve regeneration process.

## 5. Conclusions

The nanosuspension of FV seed extract enhanced the retrieval of motor and sensory functions after nerve lesion. The prepared nanosuspension enhanced the bioavailability of potent metabolites such as polyphenolic compounds. They showed a significantly higher potential for functional retrieval compared to the simple extract. They were also found to be effective in regulating glycemic levels and oxidative stress. Hence, the prepared nanoformulation served as a source of functional metabolites, increasing their bioavailability for quick recovery after sciatic nerve injury.

## Data Availability

The datasets used and analyzed during the current study are included in this manuscript.

## Abbreviations

PNI: peripheral nerve injury; HPL: hot plate latency; TAC: total antioxidant capacity; TOS: total oxidant status; TEAC: Trolox equivalent antioxidant capacity; ABTS: 2, 2′-azinobis 3 ethylbenzothiazoline-6-sulfonate.
